# Assessment of mothers’ satisfaction towards child vaccination service in South Omo zone, South Ethiopia region: a survey on clients’ perspective

**DOI:** 10.1186/s12905-024-03120-0

**Published:** 2024-05-09

**Authors:** Teshale Fikadu, Zeleke Gebru, Getachew Abebe, Selamnesh Tesfaye, Eshetu Andarge Zeleke

**Affiliations:** 1 School of Public Health, College of Medicine and Health Science, Arba Minch University, Arba Minch, Ethiopia; 2 Department of Clinical Anatomy, College of Medicine and Health Science, Arba Minch University, Arba Minch, Ethiopia; 3 Arba Minch College of Health Sciences, Arba Minch, Ethiopia; 4 College of Medicine and Public Health, Flinders University, Flinders Health and Medical Research Institute, Adelaide, South Australia

**Keywords:** Client’s perspective, Childhood vaccination, South Omo zone, Ethiopia

## Abstract

**Background:**

Even though childhood vaccination is a common and cost-effective public health intervention in preventing and reducing childhood disease and death, significant numbers of children do not complete vaccination within the first year of life. Studies indicated that user satisfaction influences service utilization and used as a key indicator of quality care. However, evidence on the level of mothers’ satisfaction with immunization service are limited in urban and accessible places and not well investigated among remote and pastoral communities. As such, this study aimed to address this gap and investigated mothers’ satisfaction towards child vaccination in a pastoralist and agrarian community of the South Omo zone in Southern region of Ethiopia.

**Methods:**

An institution-based cross-sectional study was conducted among 1221 randomly selected mothers with children eligible for childhood vaccination using a structured, pretested, and interviewer-administered questionnaire. Maternal positive evaluations of the overall vaccination service were measured using 5-point Likert scale questions. Data were entered into Epi data version 3.5.1 and analyzed using IBM SPSS statistical package version 25. Exploratory factor analysis was used for Likert scale questions to extract factor scores which facilitate treatment of variables as continuous for further analysis. Bivariate and multivariable logistic regression analysis was employed to identify factors associated with the outcome variable. A P-value < 0.05 and adjusted odds ratio with 95% CI respectively were used to declare statistical significance and degree of association.

**Result:**

A total of 849 (69.53%) study participants were satisfied with the vaccination care provided for their children. Factors associated with mother’s satisfaction with child vaccination care include maternal age less than 30 years (AOR = 2.12; 95% CI = 1.61–2.79), infants age between 8 and 12 months (AOR = 1.83; 95% CI = 1.28, 2.62), not having history of adverse events following immunization (AOR = 1.57; 95% CI = 1.01–2.45), having 1 child under the age of 5 years (AOR = 1.34; 95% CI = 1.02–1.76), waiting 30 min or less to get the service (AOR = 1.39; 95% CI = 1.05–1.85), traveling 30 min or less to the vaccination center (AOR = 1.46; 95% CI = 1.08–1.98), having poor knowledge about the importance of vaccination (AOR = 1.51; 95% CI = 1.06–2.16), and having moderate knowledge about the importance of vaccination (AOR = 1.52; 95% CI = 1.06–2.18).

**Conclusion:**

Interestingly, mothers’ satisfaction with their children’s vaccination service was relatively higher in a predominantly pastoral community compared with most of previous studies conducted in Ethiopia. Maternal and child age, number of children under the age of 5 years, history of adverse events following immunization, distance to the vaccination center, waiting time to get service and maternal knowledge were factors significantly associated with mothers’ satisfaction. Proactive measures with focus on increasing access to vaccination service, improving waiting time and raising awareness among mothers were recommended.

**Supplementary Information:**

The online version contains supplementary material available at 10.1186/s12905-024-03120-0.

## Background

More than 3 million and an estimated 472,000 children under the age of five years die each year worldwide and in Ethiopia respectively from vaccine preventable diseases [1]. In 2016, 19.5 million children did not receive the third dose of diphtheria-pertussis-tetanus (DPT_3_) worldwide with 5% dropping out [2, 3] and 5.9 million not completing vaccination in the first year of their life. Of 12.9 million (66%) eligible children who did not get any diphtheria-pertussis-tetanus (DTP) doses, 11.8 million (61%) lived in 10 countries including Ethiopia, which shared about 4% of those who did not receive DPT_3_ [2, 4, 5].

As a popular and cost-effective public health intervention in reducing childhood disease and death, childhood vaccination can reduce 2 to 3 million child deaths every year in the world [2, 6]. However, the global vaccination coverage remains at 86% [3, 7].

Vaccination coverage in hard-to-reach areas of Ethiopia was found to be low. Pentavalent vaccine coverage in Somali and Afar regions was reported as 30.7% and 45.6% respectively which is much lower than the national coverage of 65.7% [8]. In pastoralist areas where the health infrastructure is weak and the population tend to be sparse, the health care status also remains low in terms of service quality including immunization [9]. As a result, increasing immunization coverage and maintaining the quality of services has paramount importance in improving service delivery [10].

Even though sustainable access to quality supply, strong vaccination and a well-functioning health system are among the six strategies used to reach every community to benefit from vaccination [11], through observation, these are not practically fulfilled in most health facilities. Besides, trained health professionals play a key role in providing better health services, including vaccination [12]. However, a study recently conducted in central Ethiopia region in the Gurage zone revealed that 66.8% of health professionals working at vaccination rooms did not receive an in-service training on vaccination and its management [11]. Moreover, 48.7% and 32.2% of health professionals of Gurage and Bale zones in the central Ethiopia and Oromia regions respectively had unsatisfactory knowledge of cold chain management [11, 12].

Parental decision on childhood vaccination varies from refusal, intentional procrastination, and selective avoidance to full compliance with entire vaccinations [13]. Current evidence from Cyprus indicated that vaccine hesitancy has led to lower parental satisfaction and vaccination coverage, consequently causing outbreaks of vaccine-preventable diseases [13]. A World Health Organization (WHO) analysis found that high maternal and caregiver satisfaction contributes to increased vaccination compliance, which in turn increases the number of children who are fully vaccinated [14].

Client satisfaction is a basic component of health services and is directly related to the utilization of health services [15]. Studies so far documented varying degrees of maternal satisfaction with vaccination services; as low as 19.4% in Nigeria to 97% in Timor [4, 16–25]. Regarding factors known to affect mothers’ satisfaction, accessibility of service centers, waiting time, awareness about vaccination, politeness of the service providers, having information about vaccination, maternal education, parental knowledge, and distance of the vaccination site were factors influencing the maternal satisfaction towards vaccination services [1, 4, 14, 26–28].

Most literature categorize the reason for incomplete or no vaccination as a problem related to parents, the health care system, and healthcare providers [2, 6, 29–31]. In addition to maternal satisfaction, maternal knowledge is the main parental related determinant of vaccination service. Previous studies indicate the proportion of mothers with poor knowledge were 30.5% in Egypt, 36% in Cyprus, 39.91% in Debre Tabor Ethiopia, 42.5% in Malawi, 74.3% in Kombolcha Ethiopia, and 78% in Pakistan [13, 32–36].

Comprehensive knowledge on perceived maternal satisfaction towards vaccination service helps for the development of a new strategy to achieve herd immunity [13]. Despite implementing different high-impact interventions to reach every child, a significant number of children have not completed their vaccination at the recommended age perhaps partly due to low maternal satisfaction. Even though there were studies on maternal satisfaction in the urban areas [1] and central areas of the agrarian community [11, 12, 33, 37, 38], there is a lack of evidence on client perspective from a pastoralist community, one being the rural and remote areas of Southern Ethiopia.

Thus, this study aimed to assess client perspective on mothers’ satisfaction towards child vaccination service which will help improve vaccination coverage in the study area which in turn would be important to realize the sustainable development goal of leaving no one behind from health care.

## Methods and materials

### Study setting

The study was conducted among primary health care units (PHCUs) in the South Omo zone. The main town of South Omo zone, Jinka is located 555 km south of Addis Ababa, the capital city of Ethiopia. The zone has a total population of 766,749 in the year 2019/2020 as projected from the 2007 Ethiopian census. There are 3 hospitals, 34 health centers, and 226 health posts providing vaccination services for the population in the zone. This study was conducted in the five selected districts of the South Omo Zone which collectively have 1 hospital, 20 health centers and 130 health posts. PHCUs consist of health centers and health posts which serve an estimated 25,000 and 5, 000 people in their catchment areas respectively [39].

### Study design and period

An institution-based cross-sectional study was conducted from November 01 to December 30, 2020.

### Study population

All mothers or caregiver with eligible children for child vaccination (age less than 2 years) who visit health care facility for vaccination were the source population and sampled mothers or caregiver with eligible children for child vaccination were the study population Eligible children who came for vaccination with a caregiver under the age of 18 years and eligible children who were not vaccinated on the day of data collection due to different reasons were excluded from the study.

### Sample size of the study

The sample size was determined using two-population proportion formula of equal sample size for the two groups using Epi-Info7 statistical software version 7.2.3 assuming a 95% confidence interval, 80% power, 61.4% expected proportion of mothers satisfied by waiting more than 30 min to get the service (proportion among exposed), and 49.8% expected proportion of mothers satisfied by waiting less than 30 min to get the service (proportion among unexposed), a design effect of 2 and 10% potential non-response. Based on the above assumptions, the sample size calculated was 1262. Among variables considered, waiting time was selected as a variable in sample size estimation because of yielding maximum sample size compared with other potential variables [21].

### Sampling techniques

A multi-stage sampling technique was employed. Five districts were selected out of eight districts found in the zone using a lottery method. Among 151 health facilities (1 hospital, 20 health centers and 130 health posts) found in the districts selected for the study, one third (50 health facilities: 7 health centers and 43 health posts) were selected using computer generated random selection technique.

Number of eligible children (age less than 2 years) expected to be vaccinated at each selected health facility in 2019/2020 were identified and samples were allocated in proportion to the number of eligible children for vaccination. The required number of participants allocated to each healthcare facility were selected by systematic random sampling after the sampling interval (K) was calculated.

K was calculated for each health facilities after identifying average number of children vaccinated in the previous 3 months.

$$K=\frac{sample\;size\;allocated }{average\;number\;of\;monthly\;vaccinated\;children}$$

The first participant was selected randomly using vaccination registration number. Then, every k^th^ participant from each healthcare facility was enrolled until the required samples were obtained.

### Measurement

Mother’s satisfaction with vaccination service was measured using 13 (5-point Likert scale) questions. The questions were adapted from different literatures [2, 3, 6, 18, 20, 32, 34, 35, 40–43]. Factor mean was computed for each participant and participants with a score equal to or above the factor were considered as satisfied with the vaccination service; otherwise, they were considered as unsatisfied with the vaccination service. The questions address the level of maternal positive evaluation on time taken to receive service, distance to access the vaccination site, hygiene and sanitation of the equipment, courtesy and respect, politeness of the provider, skills of the provider, behavior of the provider, ways of greeting, method of providing the vaccine, measure used to keep confidentiality and trust, vaccination schedule, and the overall vaccination service.

Maternal knowledge about childhood vaccination was assessed using 9 (yes/no) questions developed from other similar studies [1–3, 5, 24–26]. The questions include maternal awareness of vaccine-preventable diseases, childhood immunization schedules, and the importance of vaccination in reducing child morbidity and mortality. The response “No” was recorded as “0 = not having awareness” and “Yes” was recorded as “1 = having awareness” with a maximum score of 9 and a minimum score of 0. Based on Bloom’s cut-off point for knowledge, those who answered 5 or less questions correctly were considered as having “poor knowledge”, those who answered 6 to7 questions correctly were considered as having “moderate knowledge”, and those who answered 8 to 9 questions correctly were considered as having “good knowledge” [44].

### Study variables

#### Dependent or outcome variable

Maternal satisfaction towards child vaccination service.

#### Independent or explanatory variables

Socio demographic characteristics: maternal age, educational status, occupation, place of residence, family size, number of under five children, average monthly income, knowledge, age and sex of the infant.

Obstetric factors: number of ANC visit, number of PNC visit, place of delivery.

Vaccination related factors: number of visits for vaccination, receiving counseling about immunization, adverse event following immunization, receiving information about the next visit, receiving counseling about adverse event following immunization.

Provider related factors: politeness, skills, behavior, greeting, courtesy and respect of the provider.

Health facility related factors: distance to vaccination centers, cleanliness of vaccination centers, vaccination schedule, sanitation and hygiene of the equipment.

### Data collection procedures and instrument

Data were collected by using a structured, pre-tested, and interviewer-administrated questionnaire. The questionnaire was first developed in English and translated into the common local language and it was checked by language experts for consistency of translation of the language. The questionnaire was developed from similar studies [2, 3, 6, 40–43] and pre-tested on 65 mothers with vaccine-eligible children from healthy families who were not selected for the actual study. The reliability of the satisfaction questionnaire was checked using Cronbach’s alpha value that was equal to 0.85. The face validity of the questionnaire was evaluated by vaccination and research experts through carefully checking of wording of the data collection tools.

### Data processing and analysis

Data were entered into Epi-data software version 3.1 and then exported to SPSS version 25.0 statistical package for analysis. After cleaning data for missing values and inconsistencies, descriptive statistics such as frequency, mean, and percentages were computed. Exploratory factor analysis was employed using the principal components analysis (PCA) method to determine the dimension among the multiple items used to make a composite score for mothers’ satisfaction with vaccination service and maternal knowledge of vaccination, and reliability analysis was conducted to determine internal consistency of the items. Accordingly, the overall cleanness of the vaccination site and information education service were removed from the mother’s satisfaction assessment questions because of unsatisfactory communality (0.41 for the overall cleanness and 0.33 for the information education service). The PCA produced a single component that explained 80.43% of the total variance by the thirteen remaining variables. Reliability analysis showed an acceptable consistency among the thirteen variables (Cronbach’s alpha = 0.84).

Similarly, the importance of completing vaccine schedule was removed due to low communality (0.48) from maternal knowledge assessment questions. The remaining 9 items with satisfactory internal consistency (Cronbach’s alpha = 0.85) were used to score maternal knowledge.

Bivariate analysis was done and all independent variables that have an association with the mother’s satisfaction with vaccination service at a p-value less than 0.25 were included in the multivariable analysis model. Then a multivariable analysis was done using a backward stepwise selection method (backward LR) to determine independent predictors of mother’s satisfaction with vaccination service. Model fitness was checked using Hosmer and Lemeshow goodness of fitness test (*P* = 0.84). Multicollinearity among independent variables was checked using variance inflation factor criterion (VIF > 10). Odds ratio with 95% CI was used to show the degree of association between the independent variables and mother’s satisfaction with child vaccination service. Level of statistical significance was declared at a p-value of less than or equal to 0.05.

## Result

### Socio-demographic and other related characteristics

A total of 1221 mothers with children eligible for vaccination participated in this study, with a response rate of 96.8%. The mean age of the mothers and children was 29 years (*±* 5.70) and 5 months (*±* 3.30) respectively. Most of the study participants, 771(63.14%) were under the age of 30 years, and 935(76.57%) lived in rural areas. Around half of the participants, 616(50.45%) had no formal education and 608(49.97%) were living with four or fewer family members (Table 1).

**Table 1:** Socio-demographic characteristics of the study participants with their children in South Omo zone, 2020

Variables	Category	Number	Percent
Maternal age	Less than 30 years	771	63.14
	30 years and more	450	36.85
Maternal educational status	No formal education	616	50.45
	Grade 1 to 8	333	27.27
	Grade 9 and above	272	22.27
Maternal occupation	Housewife	255	20.88
	Farmer	540	44.22
	Pastoralist	204	16.70
	Governmental worker	178	14.57
	Others	44	3.60
Place of residence	Urban	286	23.42
	Rural	935	76.57
Family size	<= 4	608	49.79
	5 to 6	382	31.28
	Seven and more	231	18.91
Average monthly income (ETB)	Less than 2000	353	28.91
	2000 to 4000	429	35.13
	4000 and more	439	35.95
Age of the infant	0 to 4 months	624	51.10
	4 to 8 months	318	26.04
	8 to 12 months	279	22.85
Sex of the infant	Male	650	53.23
	Female	571	46.76
Maternal knowledge	Having poor knowledge	470	38.49
	Having moderate knowledge	493	40.37
	Having good knowledge	258	21.13

### Vaccination and health-service uptake history

Most of the participants 670 (54.87%) had 1 child under the age of 5, 659 (53.97%) attended the recommended ANC, 905(74.11%) traveled 30 min and less to the vaccination center, 774(63.39%) waited 30 min to get vaccination service, 787 (64.45%) were informed about adverse events following immunization (AEFI) and 727 (59.54%) got sufficient information about the importance of vaccination (Table 2).

**Table 2:** Vaccination and health service characteristics of the study participants with their children in South Omo zone, 2020

Variables	Category	Number	Percent
Frequency of visit for vaccination	First visit	159	13.02
	Second visit	375	30.71
	Third visit	409	33.49
	Fourth and above visit	278	22.76
History of adverse events following immunization	Yes	100	9.42
	No	962	90.58
Types of complications after immunization reported by mothers or caregivers.	Fever	14	14.00
	Injection site abscess	72	72.00
	Rash	14	14.00
Number of children aged less than five years	One	670	54.87
	Two	551	45.13
Number of ANC visit	No visit at all	38	3.11
	One	12	0.98
	Two	135	11.06
	Three	377	30.88
	Four and more	659	53.97
Traveling time to the vaccination site	less than 30 min	905	74.12
	30 min and more	316	25.88
Waiting time to get service	less than 30 min	774	63.39
	30 min and more	447	36.61
Being informed about the next visit	Yes	517	42.34
	No	704	57.66
Received education about potential adverse reactions after vaccination	Yes	787	64.46
	No	434	35.54
Received education about the importance of immunization	Yes	494	40.46
	No	727	59.54

### Maternal satisfaction on childhood vaccination service

Eight hundred forty-nine (69.53%; 95% CI = 66.95–72.12) mothers were satisfied with the vaccination care provided for their child. More than 98% of the mothers satisfied with the skill of the provider and method of vaccination. Similarly, more than 97% of the participants satisfied with accessibility of the vaccination service (Table 3).

**Table 3:** Maternal responses to items used to measure satisfaction level towards vaccination service in South Omo Zone, 2020

Maternal satisfaction level with	Very Dissatisfied		Dissatisfied	Neutral	Satisfied	Very satisfied
Waiting time to get the service	1(0.08)	1(0.08)		17(1.39)	864(70.76)	338(27.68)
Courtesy and respect of the provider	0	16 (1.31)		17(1.39)	843(69.04)	345(28.26)
The politeness of the provider	0	4 (0.33)		41 (3.36)	814 (66.67)	362 (29.65)
The way their child get vaccine service	0	0		18 (1.47)	859 (70.35)	344 (28.17)
The Skills of the provider	0	0		17 (1.39)	861 (70.52)	343 (28.09)
The behavior of health worker	0	5 (0.41)		39 (3.19)	862 (70.60)	315 (25.80)
Information and education service	0	1 (0.08)		16 (1.31)	606 (49.63)	598 (48.98)
Overall cleanliness of the vaccination site	6 (0.49)	9 (0.74)		92 (7.53)	297 (24.32)	817 (66.91)
Sanitation and hygiene of the equipment	0	2 (0.16)		0	580 (47.50)	639 (52.33)
Provider greeting	0	2 (0.16)		5 (0.41)	538 (44.06)	676 (55.36)
Measure taken to confidentiality and trust	0	4 (0.33)		24 (1.97)	546 (44.72)	647 (52.99)
The used materials for vaccination	0	0		1(0.08)	569 (46.60)	651 (53.32)
Distance traveled to vaccination center	0	0		20 (1.64)	585 (47.91)	616 (50.45)
Vaccination schedule	0	6 (0.49)		21 (1.72)	605 (49.55)	589 (48.24)
Overall vaccination service	35 (2.87)	59 (4.83)		29 (2.38)	562 (46.03)	536 (43.90)
The overall maternal satisfaction	Number	Percent				
Yes No	849 372	69.53 30.47				

### Factors associated with maternal satisfaction towards child vaccination

Among 15 candidate variables for multivariable analysis, seven were identified as independent factors affecting maternal satisfaction towards childhood vaccination service. These were maternal age, child age, number of children under the age of 5 years, traveling time to vaccination center, waiting time to get service, having history of adverse events following immunization, and maternal knowledge.

The odds of maternal satisfaction was 2 times higher among participants under 30 years compared to those who were 30 years and above (AOR = 2.12; 95% CI = 1.61–2.79). Mothers with an older infant were 1.9 times more likely to be satisfied compared to those who had younger infants (AOR = 1.83; 95% CI = 1.28, 2.62). The odds of maternal satisfaction was 1.5 times higher among mothers of children with no history of adverse events following immunization than those with a history of adverse events following immunization (AOR = 1.57; 95% CI = 1.01–2.45). Similarly, the odds of maternal satisfaction was 1.3 times higher among mothers with 1 child under 5 years older than mothers with 2 children (AOR = 1.34; 95% CI = 1.02, 1.76). Participants who waited below 30 min to get the service were 1.4 times more likely satisfied than those who waited more than or equal to 30 min (AOR = 1.39; 95% CI = 1.05, 1.85). Also, respondents who traveled less than 30 min to the vaccination-center were 1.4 times more likely to be satisfied than those who traveled more than or equal to 30 min (AOR = 1.46; 95% CI = 1.08–1.98). Mothers who had poor and moderate knowledge about the importance of vaccination were 1.5 times more likely to be satisfied than those who had good knowledge (AOR = 1.51; 95% CI = 1.06–2.16) and (AOR = 1.52; 95% CI = 1.06–2.18) (Table 4).

**Table 4:** Factors associated with maternal satisfaction toward childhood vaccination in south Omo Zone 2021

Variable	Category	Satisfied N^*o*^ (%)	Unsatisfied N^*o*^ (%)	COR(95% CI)	AOR(95% CI)
Maternal age	less than 30 years	585(68.90)	186(50.00)	2.22(1.73,2.85)**	2.12(1.61,2.79)**
	30 Years and more	264(31.10)	186(50.00)	1	1
Age of the child	0 to 4 months	407(47.90)	217(58.30)	1	1
	4 to 8 months	232(27.30)	86(23.10)	1.44(1.07,1.94)*	1.33(0.95,1.86)
	8 to 12 months	210(24.70)	69(18.50)	1.62(1.18,2.23)*	1.83(1.31,2.62)*
History of AEFI	Yes	59(8.10)	41(12.40)	1	1
	No	672(91.90)	290(87.60)	1.61(1.06,2.46)*	1.57(1.01, 2.45)*
Number of < 5 years children	One	484(57.00)	186(50.00)	1.33(1.04,1.69)*	1.34(1.01,1.76)*
	Two	365(43.00)	186(50.00)	1	1
Traveling time to vaccination site	less than 30 min	652(76.80)	253(68.00)	1.56(1.19,2.04)*	1.46(1.08,1.98)
	30 min and more	197(23.2)	119(32.00)	1	1
Waiting time to get vaccination service	less than 30 min	560(66.00)	214(57.50)	1.43(1.11,1.82)*	1.39(1.05,1.85)*
	30 min and more	289(34.00)	158(42.50)	1	1
Maternal knowledge	Having poor knowledge	332(39.10)	138(37.10)	1.38(0.99,1.90)	1.52(1.06,2.18)*
	Having moderate knowledge	353(41.60)	140(37.60)	1.45(1.05,1.99)*	1.51(1.06,2.16)*
	Having Good knowledge	164(19.30)	94(25.30)	1	1

AEFI = adverse event following immunization ** *p* < 0.01, * *p* < 0.05

## Discussion

Our study indicated that 7 out of 10 mothers / caregivers were satisfied with childhood vaccination service and nearly two third of the participants had moderate to good knowledge about childhood vaccination. Modifiable factors that determined maternal satisfaction were maternal knowledge, waiting time, accessibility, AEFI and child spacing.

About 69.53% of the study participants were satisfied with the vaccination service provided for their children which was interestingly higher satisfaction level compared with most studies conducted in Ethiopia and elsewhere. It was also comparable with some previous studies conducted in Ethiopia [27, 38]. The finding was higher than studies conducted in Pakistan, Jimma, Jiggija, Hadiya, Egypt, Nigeria, Timor, Addis Ababa, and Bahir Dar [1, 20–25, 32, 35, 45, 46]. But this finding is lower when compared with studies conducted in Ethiopia, Nigeria, Egypt and Saudi Arabia [18, 19, 23, 24, 26, 47]. Not suprisingly, the difference can be associated to differences in awareness and literacy, the subjective nature of the response, and the items used to measure the satisfaction level between the studies.

The odds of maternal satisfaction were higher among mothers of study participants aged less than 30 as compared to their counterparts. It is well-known facts that as people get older, their health care utilization, knowledge of care and their expectations increase, and as a result their satisfaction with care decreases. Women, particularly in developing countries, usually are challenged with the triple burden of responsibility which includes productive, reproductive, and community management roles which all increase with increasing age. For women, managing household tasks is culturally considered sole responsibility in many cultures. Child rearing and bearing are women’s prime responsibilities in most developing countries including Ethiopia. Community and social responsibilities which are voluntary and unpaid also are another time-consuming responsibility a woman is expected to take. All these situations affect maternal satisfaction with the service provided to their child. Besides, perception and attitude differences towards a service might be another explanation. Earlier age women are usually having positive attitudes and non-complaining behaviors toward health service and health service providers when compared with their counterparts. This is consistent with studies in Ethiopia, Saudi Arabia and Nigeria [24, 47, 48].

Mothers who had older infants were more likely to be satisfied than those who had younger infants. This might be due to the biological immaturity younger infants are characterized. As a result, every exposure to infection might result in adverse events at a young age. Women lived and shared experiences with infants at this age put them in a worrying situation during each event. As their infant’s age increases, experience with adverse effects of vaccines and women’s subsequent stress also start to decrease which in turn improves their satisfaction with the services given to a child. This is consistent with a study in Guatemala and Vietnam [4, 14].

Higher odds of being satisfied were reported among women whose child did not develop (AEFI) when compared to those who develop AEFI. Adverse event is a medical incident that takes place after immunization and is believed to be a reaction caused by the immunization such as mild inflammation, pain and redness of the vaccine at the injection site, low-grade fever, mild rash, and more commonly after the vaccination, some babies may show irritability (easy to be upset) and discomfort. When infants develop any of these, even if mild and manageable, most women get worried and unsatisfied. This is consistent with a previous study in north Ethiopia [18].

Likewise, in the present study number of under five years of children were found to be a significant factor affecting maternal satisfaction towards childhood vaccination. This is supported by a study conducted in Guatemala [4]. In many cultures in many developing countries including Ethiopia, children are considered assets. This belief demands a high number of children who can survive, and the corresponding fear becomes more when having more under five-year children.

Waiting time to get service was identified to be a significant factor affecting maternal satisfaction towards childhood vaccination. This is supported by studies conducted in different parts of Ethiopia and Nigeria [24, 26, 38, 48]. This might be commonly related to traveling time or accessibility-related challenges which are likely be the case in a pastoralist community with sparsely distributed population. Since most users travel many kilometers to get this service, they want to be served within a short time of arrival. Delays in service delivery can lead to emotional instability and dissatisfaction with the services provided. The busy life experience of women is also another explanation.

Maternal knowledge was found to be a significant factor affecting maternal satisfaction towards childhood vaccination. This is in line with findings reported from previous studies in different parts of Ethiopia [23, 35]. This might be since knowledge is a powerful predictor of attitude and satisfaction is strongly related to one’s attitude. In the current study, a higher knowledge score resulted in a less satisfaction. This can go with the bare fact that being knowledgeable about vaccination can go with increased expectations of quality service and hence a decreased satisfaction.

Traveling time to the vaccination site was also found to be a significant factor affecting maternal satisfaction with child vaccination. This is consistent with a study conducted in Ethiopia [26] and inconsistent with studies conducted in Ethiopia and Nigeria [18, 24]. This might be related to the fact that limited coverage of health care facilities is a common characteristic of the country. Thus, most mothers are forced to travel long distance to get a health service. Long exhaustive travel of users combined with long waiting time at facilities affects satisfaction. Evidence indicated that traveling long distance was one of the reasons for defaulting from vaccination [5].

## Limitations of the study

There might be recall bias during data collection by participants, especially the adverse events following immunization, and the time they spent to get the service. There might also be social desirability bias during the face-to-face interview.

## Conclusion and recommendation

This study showed that level of satisfaction was high compared with most study conducted in Ethiopia. and maternal and child age, number of < 5 years old children, adverse events following immunization, travel time to vaccination centers, waiting time to get service and mothers’ knowledge about vaccination were identified to be determinants of mothers’ satisfaction. So concerned health authorities and health professionals should strengthen the existing child spacing interventions and outreach vaccination services. Maternal education and counseling should be strengthened during the prenatal, well-baby and sick infant care. Moreover, health care providers were recommended to implement safe injection practice and to provide timely service.

### Electronic supplementary material

Below is the link to the electronic supplementary material.

Supplementary Material 1

## Data Availability

All relevant data are within the paper. However, if additional information is required, it will be provided upon request from corresponding author.
