# Supplementary material for: Assessment of mothers’ satisfaction towards child vaccination service in South Omo zone, South Ethiopia region: a survey on clients’ perspective
Source: BMC Womens Health. 2024 May 9;24:272. doi: 10.1186/s12905-024-03120-0 (PMC11080138; doi:10.1186/s12905-024-03120-0)
Supplement: Supplementary file 1 — Supplementary Material 1 [file 12905_2024_3120_MOESM1_ESM.docx]

Supporting Table 1 Mothers’ responses to knowledge and level of satisfaction questions

| **Knowledge question** | **Yes**  **Frequency (%)** | **No**  **Frequency (%)** |
| --- | --- | --- |
| Is infectious disease prevented by child vaccination? | 1086 (88.94) | 135 (11.06) |
| Is vaccination use full for children | 1102 (90.25) | 119 (9.75) |
| Does childhood vaccination cause disease | 1047 (85.75) | 174 (14.25) |
| Does Vaccinating a child with illness can get relief | 795 (65.11) | 426 (34.89) |
| Is complete childhood vaccination before first birth day helpful? | 613 (50.20) | 608 (49.80) |
| Is completing all vaccination schedules important for the child | 941(77.07) | 280 (22.93) |
| Does keeping an appointment schedule is helpful? | 688 (56.35) | 533 (43.65) |
| Does fever after childhood vaccination is sign of illness | 244 (19.98) | 977(80.02) |
| Is vaccinate a child on the first day helpful | 340 (27.85) | 881 (72.15) |
| Does vaccination used as treatment | 916 (75.02) | 305 (24.98) |
